# Functional reorganization of the visual cortex in blindness: a connectivity perspective

**DOI:** 10.3389/fnhum.2026.1884190

**Published:** 2026-08-18

**Authors:** Xiang Xiao, Yu Zhang, Jiacheng Zheng, Mengyu Tian

**Affiliations:** 1 Bay Area School of Applied Psychological Sciences, Beijing Normal University at Zhuhai, Guangdong, China; 2 College of Education for the Future, Beijing Normal University, Zhuhai, China

**Keywords:** blindness, brain connectivity, cortical plasticity, occipital cortex, visual deprivation

## Abstract

Visual deprivation provides a critical model for understanding how sensory experience and developmental constraints shape cortical organization. In blindness, the occipital cortex is recruited for tactile and auditory perception as well as language, memory, mathematical processing, and executive control. The core question is how visual cortex becomes functionally embedded in non-visual and cognitive systems, and how this embedding gives rise to particular forms of occipital recruitment. Connectivity is central to this question because cortical functions are shaped, in part, by the information a region receives, the pathways through which it exchanges signals, and its coordination with distributed systems. Connectivity therefore links developmental constraints and sensory experience to functional emergence: it specifies which signals can influence occipital regions, which networks can incorporate them, and which functional roles they may come to support. Resting-state studies show that some retinotopy-consistent features of local occipital organization persist without visual experience. At the same time, reduced homotopic and interhemispheric connectivity suggests altered coordination within the visual system. Long-range functional connectivity reveals coexisting patterns: ventral occipitotemporal regions can retain broadly sighted-like connectivity profiles, while occipital cortex also shows enhanced coupling with frontoparietal, language-related, and other higher-order networks. This large-scale network embedding provides a mechanistic account of how occipital regions can participate in higher-order cognition. Structural connectivity offers a complementary but still limited account. Most studies report alterations in visual afferent pathways, occipital white matter, posterior association fibers, and posterior callosal pathways, and these findings have often been interpreted within a deficit-oriented framework. However, structural findings alone do not yet explain how anatomical connectivity supports functional recruitment, how structural profiles relate to functional connectivity, or how structure–function coupling develops with sensory history and individual experience. Whole-brain connectome studies further point to altered network efficiency, path length, and structure–function alignment, but the mechanisms linking these features to occipital function remain unresolved. Together, current evidence suggests that connectivity should be treated as a mechanism of occipital plasticity. Future work should clarify how preserved architectural scaffolds, anatomical constraints, and experience-dependent functional coupling jointly shape the emergence of non-visual and cognitive functions in the blind occipital cortex.

## 1 Introduction

The human brain is characterized by remarkable adaptability and plasticity. A central question in neuroscience concerns how experience shapes brain structure and function, and to what extent innate predispositions constrain cortical organization. Visual deprivation provides a particularly powerful model to address this issue. In primates, more than one-third of the cortex is associated with visual processing (Felleman and Van Essen, 1991), yet in individuals who are blind from early in life, the absence of visual input leads to markedly altered developmental trajectories of the “visual” cortex. At the same time, blind individuals rely on non-visual modalities to acquire information and guide behavior, forming highly specific experiential structures, such as tactile Braille reading and non-visual spatial navigation.

These observations raise a key question: how is the visual cortex functionally organized in the absence of visual experience, and what neural mechanisms give rise to this organization? A growing body of work suggests that brain function cannot be understood in terms of isolated regions, but instead emerges from interactions within distributed networks (Thiebaut De Schotten and Forkel, 2022). In this perspective, patterns of connectivity play a central role in shaping cortical organization, as they constrain the information a region can receive and process, and thereby determine its functional profile. Accordingly, understanding how the visual cortex reorganizes in blindness requires examining the structural and functional connectivity of the visual cortex across multiple scales. In this review, we approach this question from a connectivity perspective, focusing on both structural and functional connections to characterize the organization of the visual cortex in blindness.

## 2 Functional reorganization of the visual cortex

A substantial body of research has shown that the occipital cortex in blind individuals can be recruited for processing information from non-visual modalities (Merabet et al., 2004; Pascual-Leone et al., 2005), although the mechanisms underlying such reorganization remain debated.

The classical crossmodal, or metamodal, theory proposes that sensory cortices implement modality-invariant cognitive functions that can operate on inputs from different sensory modalities (Pascual-Leone and Hamilton, 2001). Consistent with this account, studies have shown that the occipital cortex in blind individuals preserves functional organizational principles similar to those observed in sighted individuals. For example, the dorsal occipital cortex, typically involved in visual spatial processing in sighted individuals, has been shown to support auditory spatial processing in blind individuals (Collignon et al., 2011). Similarly, the middle temporal motion complex (MT), which is selectively responsive to visual motion in sighted individuals, responds to tactile and auditory motion in blindness (Ricciardi et al., 2007; Wolbers et al., 2011b). A similar pattern is observed in ventral visual cortex. When stimuli such as faces, tools, or scenes are presented auditorily, blind individuals exhibit spatial patterns of category-selective activation that closely resemble those of sighted individuals (Arbel et al., 2022; He et al., 2013; Mattioni et al., 2020, 2022; van den Hurk et al., 2017; Wang et al., 2015). Likewise, tactile presentation of faces and scenes activates canonical category-selective regions, including the fusiform face area (FFA) and the parahippocampal place area (PPA), in congenitally blind individuals (Ratan Murty et al., 2020; Wolbers et al., 2011a). The visual word form area (VWFA) has also been implicated in tactile word-form processing during Braille reading (Haupt et al., 2024; Matuszewski et al., 2025; Reich et al., 2011), although this interpretation remains debated (see Tian et al., 2023 for alternative accounts). In addition, the inferolateral occipitotemporal cortex (ILOTC) exhibits selectivity for shape representation, rather than conceptual information, in both blind and sighted individuals (Xu et al., 2023).

Building on these findings, the modality-independent, task-specific framework has been proposed to account for the preserved functional organization of sensory cortices in the absence of their typical inputs (Amedi et al., 2017; Heimler et al., 2015). This framework suggests that cortical organization is constrained by two complementary principles. First, local circuits are sensitive to task-distinctive computational features, such as shape, spatial location, or motion, in a manner that is largely independent of sensory modality. Second, cortical regions are embedded within pre-existing patterns of connectivity that link them to distributed networks supporting specific functions, thereby biasing their functional recruitment during development. Such connectivity biases are already evident early in life and are thought to be shaped, at least in part, by innate genetic constraints (Barttfeld et al., 2018; Li et al., 2020; Petersen et al., 2024). These principles provide a parsimonious account of functional reorganization in blindness. They explain why occipital regions can be recruited by non-visual inputs while still exhibiting functional preferences similar to those observed in sighted individuals: sensory-deprived cortices continue to process the same type of information, but through inputs from alternative modalities, within the constraints imposed by pre-existing patterns of connectivity.

In contrast, a growing body of research has shown that the occipital cortex in congenitally blind individuals can also be recruited for higher-order cognitive functions that differ substantially from visual processing at both cognitive and evolutionary levels. The occipital cortex in congenitally blind individuals is engaged during auditory language tasks involving different levels of linguistic processing, including lexical decision, semantic judgment, and verb generation from nouns, whereas similar tasks do not recruit the visual cortex in sighted individuals (Amedi et al., 2004; Burton et al., 2003, 2012; Röder et al., 2002). This engagement is not merely a general task effect, as occipital responses are modulated by specific linguistic properties of the stimuli, with stronger responses to meaningful words and to sentences with increased syntactic complexity (Bedny et al., 2011; Lane et al., 2015; Röder et al., 2002). Importantly, greater occipital activation is associated with better behavioral performance in syntactic tasks (Lane et al., 2015). In addition to language, the “visual” cortex in congenitally blind individuals is also recruited during auditory mathematical processing and shows sensitivity to task difficulty (Amalric et al., 2018; Kanjlia et al., 2016). Furthermore, several studies have reported that the occipital cortex in congenitally blind individuals is sensitive to cognitive control demands and multiple-demand processing, exhibiting functional characteristics similar to those of the frontoparietal control network (Duymuş et al., 2024; Kanjlia et al., 2021). Functional differentiation is also evident across occipital subregions, with partially distinct regions recruited across higher-order domains such as language, executive control, and long-term memory retrieval (Abboud and Cohen, 2019). Given that language, mathematics, and executive control are typically supported by transmodal association cortices in sighted individuals, their recruitment of occipital cortex in congenital blindness is difficult to explain as a simple crossmodal substitution of non-visual inputs into pre-existing visual computations, or as sensitivity to perceptual dimensions such as spatial location, motion, shape, and object category.

This pattern of higher-order recruitment has been interpreted in terms of the cognitively pluripotent hypothesis, which proposes that the cortex is initially endowed with substantial functional flexibility, allowing it to support a wide range of cognitive functions during development (Bedny, 2017). In this framework, cortical specialization is driven by information a region receives during development, which is itself constrained by connectivity and experience. In the absence of bottom-up visual input from the lateral geniculate nucleus, input from higher-cognitive networks may dominate activity in the developing visual cortex and lead it to assume higher-cognitive functions. The more recent connectivity-constrained experience-dependent account builds on this view by further emphasizing the interaction between pre-existing long-range connectivity, altered sensory experience, and behavioral demands in shaping the functional profile of occipital cortex in congenital blindness (Saccone et al., 2024). Accordingly, long-range connections between the occipital cortex and other brain regions are thought to play a critical role in functional reorganization, as they determine the type and range of information that a given cortical region can receive and transmit within large-scale neural circuits (Passingham et al., 2002; Thiebaut De Schotten and Forkel, 2022).

The cognitively pluripotent account further emphasizes that cortical regions have broad functional potential early in development. Within this framework, greater occipital involvement in higher-order cognitive functions is expected in congenital or early blindness than in adult-onset blindness. Several empirical findings are consistent with this developmental expectation. Compared with adult-onset blind individuals, congenitally blind individuals show stronger or more differentiated occipital responses during spoken-language processing, mathematical reasoning, and naturalistic auditory stimulation (Bedny et al., 2012; Kanjlia et al., 2019; Musz et al., 2022; Pant et al., 2020). Effects of blindness onset have also been reported for non-visual perceptual functions. Auditory spatial selectivity in dorsal occipital cortex and auditory motion representations in hMT + are more evident following congenital or early blindness than after later visual loss (Collignon et al., 2013; Jiang et al., 2016). However, the influence of blindness onset varies across functional outcomes. Later visual loss can also be associated with functionally specific occipital recruitment, as shown for voice-identity processing in fusiform cortex (Hölig et al., 2014), and early- and late-blind groups show broadly similar changes in sound-category representations in ventral occipitotemporal cortex (Mattioni et al., 2022). Current evidence suggests that blindness onset constrains some forms of occipital functional specialization more strongly than others.

Despite their different accounts of cortical plasticity, both frameworks emphasize connectivity as a key constraint on regional function. This shared emphasis aligns with a broader view of brain organization, in which cortical functions emerge from interactions within distributed networks rather than from isolated regions alone (Thiebaut De Schotten and Forkel, 2022). In neuroimaging research, connectivity is typically described in terms of structural and functional connectivity. Structural connectivity denotes the pattern of white matter pathways linking different brain regions and is typically estimated using diffusion-weighted imaging (DWI). DWI-based approaches broadly fall into two categories. One quantifies microstructural properties of white matter, such as fractional anisotropy (FA), which index features including fiber organization and integrity. The other reconstructs white matter tracts to derive inter-regional connectivity matrices, capturing large-scale patterns of structural connectivity (Zhang et al., 2022). Functional connectivity, in contrast, denotes the temporal co-variation of neural activity across brain regions. In fMRI research, it is typically estimated from blood-oxygen-level-dependent (BOLD) signals, most often as temporal correlations between regions (Biswal et al., 1995). Importantly, functional connectivity does not necessarily imply a direct anatomical connection between regions, as temporal correlations may reflect indirect interactions mediated by intermediate regions or common inputs.

The two theoretical accounts lead to different expectations regarding occipital connectivity in blindness. Under the modality-independent task-specific framework, occipital connectivity is expected to show substantial similarity to that observed in sighted individuals despite the absence of visual experience. This applies both to the internal organization of the occipital cortex and to its connectivity with other functional systems. These connectivity patterns are therefore expected to support task-specific occipital responses in blind individuals that resemble the corresponding responses in sighted individuals. In contrast, the cognitively pluripotent hypothesis predicts that occipital reorganization in blindness should be supported by the integration of occipital cortex into large-scale cognitive networks. On this account, the higher-order recruitment described above would be expected to rely on strengthened or redistributed functional connectivity with networks that normally support the relevant computations, particularly frontoparietal control and language-related networks. The key prediction is that occipital cortex becomes more strongly integrated with frontoparietal control and language-related networks that can support its altered functional roles. These theoretical predictions make occipital connectivity a critical testing ground for understanding visual cortical reorganization in blindness.

These contrasting accounts also form part of a broader debate over what should qualify as cortical reorganization. Makin and Krakauer (2023) defined reorganization in a strong mechanistic sense as a qualitative change in the functional identity or computational capacity of a cortical area. Under their framework, evidence for reorganization would require the convergence of three conditions: the emergence of a novel input, or a novel output in the case of motor cortex; the emergence of a local computation not present in the typical brain; and the coherent downstream readout of these changes to support a novel, behaviorally relevant function. They argued that the current evidence from blindness does not establish this combination. For example, non-visual responses in V1 may reflect the unmasking or gain modulation of pre-existing inputs rather than the acquisition of novel inputs or computations. They further questioned whether the available evidence demonstrates genuinely novel higher-order cognitive processing in V1, given the possible confounds in many complex tasks. For lateral and ventral occipitotemporal cortex, they proposed that crossmodal responses may reflect either multisensory capacities already present in sighted individuals or input-agnostic capacities, while preserving the region’s pre-existing domain preferences and representational organization (Makin and Krakauer, 2023). From this perspective, many findings conventionally described as cortical reorganization may instead reflect quantitative plasticity within a pre-existing functional architecture.

Makin and Krakauer’s (2023) requirement of novel inputs or outputs, novel computations, and a behaviorally relevant contribution of those computations provides a reasonable criterion for establishing cortical reorganization in the strict sense. However, the unmasking or gain modulation of pre-existing inputs, or the expression of multisensory or input-agnostic capacities, does not appear sufficient to account for the task-selective or difficulty-sensitive responses observed in the occipital cortex during some higher-order cognitive tasks. We nevertheless retain the term functional reorganization because it is conventional in the blindness literature and is used here in a broader, descriptive sense. Specifically, it refers to changes in the conditions under which occipital regions are recruited and in their functional interactions with other brain systems following the absence or loss of visual experience. This usage does not assume that native cortical computations have been replaced, that entirely new computations have been conclusively demonstrated, or that new anatomical connections have formed. With these theoretical distinctions and the scope of functional reorganization clarified, the present review focuses on how different forms of connectivity relate to the functional profile of the occipital cortex in blindness. Existing studies have approached this issue from several directions, including local functional connectivity within the occipital cortex, long-range functional connectivity with distributed brain networks, and structural connectivity along visual and association pathways. The following sections review evidence from these different lines of work.

## 3 Local organization of functional connectivity in the occipital cortex

Evidence relevant to these predictions first comes from studies of local functional connectivity within the occipital cortex. In sighted individuals, resting-state functional connectivity within the visual cortex generally follows retinotopic principles, with systematic variation along dimensions such as eccentricity and polar angle (Heinzle et al., 2011; Raemaekers et al., 2014; Wandell et al., 2007). Several resting-state fMRI studies show that patterns of intrinsic functional connectivity within the occipital cortex of blind individual preserves key features of retinotopic organization observed in the sighted. Functional connectivity remains systematically differentiated along the eccentricity axis, with distinct patterns between regions representing central and peripheral visual fields, and critically, polar angle reversals at the V2/V3 border are preserved. Connective field (CF) modeling, originally introduced by Haak et al. (2013), extends the receptive-field concept from stimulus space to cortical connectivity by estimating how activity at each location in a target visual area can be predicted from a spatially weighted pattern of activity in a source area. When applied to resting-state fMRI, CF modeling can characterize the intrinsic topographic organization of interareal functional connectivity, while Bayesian implementations permit the associated uncertainty of CF estimates to be quantified (Invernizzi et al., 2021). Bock et al. (2015) used CF modeling to show that retinotopy-consistent connectivity between V1 and V2/V3 was preserved in early blind and anophthalmic individuals, converging with findings obtained using other resting-state approaches (Butt et al., 2015; Striem-Amit et al., 2015). At a finer scale, spontaneous BOLD correlations within V1 show highly similar patterns in blind and sighted individuals, decreasing with cortical surface distance, suggesting that local functional organization is largely independent of visual experience (Butt et al., 2013).

At the same time, converging evidence indicates that intrinsic functional connectivity within the occipital cortex is selectively altered in blindness. In the sighted visual cortex, functional connectivity reflects both direct anatomical links between adjacent visual areas and coupling between homotopic regions that lack direct structural connections but share similar retinotopic representations (Heinzle et al., 2011; Innocenti and Price, 2005; Lyon and Kaas, 2002). In contrast, blind individuals exhibit increased functional connectivity between hierarchically adjacent visual areas that are linked by direct anatomical connections (Heine et al., 2015; Qin et al., 2013). At the same time, they show reduced connectivity between homotopic regions with similar eccentricity representations but without direct anatomical connections, such as V2v and V2d, as well as decreased interhemispheric connectivity across occipital regions, including V1 and lateral visual areas such as V2–V4, V3A, and LOC (Abboud and Cohen, 2019; Butt et al., 2015; Heine et al., 2015; Hou et al., 2017; Qin et al., 2013; Watkins et al., 2012). Reduced interhemispheric homotopic connectivity is also associated with blindness duration (Hou et al., 2017).

How these preserved and altered features depend on the timing of visual loss has been examined through direct comparisons between congenital and later-onset blindness and through studies of progressive visual loss acquired in adulthood. Butt et al. (2015) found that the spatial extent of hierarchical and homotopic correlations was increased in congenital blindness, whereas later-onset blind individuals were comparable to sighted controls. Correlation amplitude, however, did not differ significantly between the congenital and later-onset blind groups. A related pattern has been observed in primary open-angle glaucoma (POAG), which provides a model of spatially restricted and progressive loss of visual input. Using population receptive field (pRF) mapping and micro-probing-based visual-field reconstruction, Carvalho et al. (2022) found that V1, V2, and V3 retained their coarse retinotopic organization despite glaucomatous visual-field loss, whereas BOLD responses and local pRF size and position showed heterogeneous, participant-specific deviations relative to controls with matched simulated scotomas (Carvalho et al., 2020, 2022). A recent preprint using Bayesian CF modeling likewise found preserved coarse retinotopic organization but smaller CFs in POAG, most consistently for V1-to-V2 connections in both stimulus-driven and resting-state data, with broadly similar but less consistent evidence for V1-to-V3 connections. In the stimulus-driven condition, matched simulated scotomas did not reproduce the reduction in CF size, and CF size was not clearly related to disease severity (Invernizzi et al., 2025).

Overall, in the absence of visual experience, the occipital cortex in blind individuals neither uniformly preserves nor entirely loses its original organization. Instead, features consistent with retinotopic organization are selectively retained, primarily in connections supported by direct anatomical pathways, whereas connectivity patterns lacking such anatomical support are systematically reduced (Butt et al., 2013; Striem-Amit et al., 2015). This pattern may suggest that connectivity without direct anatomical substrates is more dependent on visual experience for its maintenance, including components related to interhemispheric integration. The preservation of retinotopy-consistent connectivity is in line with the biased connectivity principle proposed in modality-independent, task-specific accounts of cortical organization. However, whether preserved or altered, these local organizational features alone are insufficient to account for the range of functional changes observed in the occipital cortex of blind individuals. Understanding these changes requires moving beyond local organization to examine how the occipital cortex interacts with distributed brain networks at a larger scale.

## 4 Long-range functional connectivity of the occipital cortex

Task-based fMRI studies in congenitally blind individuals reveal two distinct but coexisting patterns of occipital function. First, some occipital regions appear to process information from non-visual sensory modalities while preserving functional organization comparable to those observed in sighted individuals. For example, ventral visual cortex retain category-selective response profiles comparable to those observed in sighted individuals. Second, the occipital cortex is recruited for higher-order cognitive functions, including language, mathematical processing, and executive control, which are typically supported by frontoparietal association cortices in the sighted brain. Notably, this latter pattern reflects the engagement of occipital cortex in functions beyond its canonical perceptual role, rather than mere shift of the processing modality. These findings motivate examining how the occipital cortex interacts with multiple cognitive systems. Accordingly, a substantial body of work has investigated resting-state functional connectivity between occipital cortex and distributed brain regions in congenitally blind individuals, focusing on the connectivity profiles of these interactions and their alignment with specific cognitive systems.

Relative preservation of long-range functional connectivity is most evident in category-selective regions of the ventral occipitotemporal pathway. In the fusiform face area (FFA), resting-state functional connectivity predicts the individual-level topography of face selectivity in both blind and sighted individuals. Importantly, this connectivity–function mapping generalizes across groups: models trained in one group can predict face-selective patterns in the other (Ratan Murty et al., 2020). Similarly, occipitotemporal and temporal regions involved in unique-entity semantics show largely comparable functional connectivity patterns in blind and sighted individuals (Wang et al., 2016). Connectivity between occipital and frontoparietal regions involved in object shape processing is likewise highly consistent between early blind and sighted individuals (Xu et al., 2023). Together, these findings suggest that some long-range connectivity profiles of ventral occipitotemporal cortex are preserved despite the absence of visual input, in line with the idea that these regions are partly constrained by connectivity architecture established early in development.

However, this preservation of connectivity is spatially heterogeneous across the occipital cortex. A study comparing voxel-wise functional connectivity fingerprints between blind and sighted individuals found that anterior occipital regions—including anterior medial ventral occipitotemporal cortex and parts of the lateral temporal cortex—show a high degree of similarity across groups. In contrast, posterior occipital regions, such as early visual cortex and posterior lateral fusiform gyrus, exhibit markedly lower between-group similarity (Wang et al., 2015). This pattern indicates that different occipital regions may follow distinct developmental trajectories depending on experience, with early visual cortex showing greater divergence between groups than higher-order visual areas.

Beyond these similarity-based findings, other studies have focused on how occipital connectivity differs between blind and sighted individuals. Studies using both whole-brain and ROI-based approaches have consistently shown that, compared with sighted controls, blind individuals exhibit reduced functional connectivity between occipital cortex and other non-visual sensory cortices, such as somatosensory and primary auditory cortex (Liu et al., 2007; Qin et al., 2013; Yu et al., 2008). This reduction is observed in primary visual cortex as well as in parts of higher-order visual areas. In contrast, blind individuals show increased functional connectivity between occipital and prefrontal regions, particularly with the language-related left inferior frontal gyrus and adjacent regions (Butt et al., 2013; Heine et al., 2015; Liu et al., 2007; Striem-Amit et al., 2015). In line with this pattern, independent component analysis of resting-state functional connectivity showed that lateral occipital cortex was incorporated into a left-lateralized language-related component in blind individuals, but not in sighted individuals (Watkins et al., 2012). Other work reported increased occipital connectivity with a broader set of prefrontal regions, including left premotor cortex, medial prefrontal cortex, and portions of the inferior frontal gyrus (Deen et al., 2015). Task-based data from the same study indicated that only a subset of inferior frontal areas showed language selectivity, whereas most regions were engaged by working memory or executive control demands (Deen et al., 2015).

These broad whole brain level findings suggest that occipital connectivity in blindness is shifted toward prefrontal and other higher-order systems, but they do not specify how this altered connectivity relates to particular cognitive functions. A more targeted approach has therefore defined occipital regions based on task activation and then examined whether these regions preferentially connect with networks supporting the corresponding cognitive domains. Occipital regions that respond to language tasks in blind individuals preferentially connect with inferior frontal language areas, whereas those that respond to mathematical tasks show stronger connectivity with the intraparietal sulcus and dorsal prefrontal regions supporting numerical processing (Bedny et al., 2011; Kanjlia et al., 2016). Similarly, occipital regions that respond to executive control demands—particularly those sensitive to conflict—exhibit stronger resting-state functional connectivity with prefrontal control-related regions (Kanjlia et al., 2021). A study using multiple tasks within the same participants further examined functional differentiation within the occipital cortex and its corresponding long-range connectivity patterns (Abboud and Cohen, 2019). Across semantic processing, verb generation, executive control, and long-term memory retrieval tasks, distinct task-related regions could be identified within the occipital cortex, despite partial overlap among them. Resting-state analyses showed that these task-defined occipital subregions exhibited functional dissociable connectivity patterns with frontal and parietal cortices (Abboud and Cohen, 2019). These findings provide further support for the cognitively pluripotent account by specifying how functional connectivity contributes to the involvement of occipital cortex in multiple higher-order cognitive functions. Rather than indicating only a broad shift toward higher-order systems, they suggest that different occipital subregions form relatively stable long-range connectivity patterns with distinct cognitive networks. Strengthened, task-specific connectivity with frontal and frontoparietal systems may enable occipital cortex to participate in the corresponding cognitive functions.

Blindness onset also influences long-range functional connectivity between occipital cortex and higher-order cortical regions. Butt et al. (2013) found stronger functional connectivity between V1 and the left inferior frontal language region in congenitally or early blind individuals than in later-onset blind individuals. Bedny et al. (2010) found reduced connectivity between MT/MST and early visual cortex in both blind groups, whereas enhanced connectivity between MT/MST and lateral prefrontal cortex was observed only in congenital blindness. Kanjlia et al. (2019) likewise found that connectivity between task-defined occipital regions and frontal or parietal regions involved in mathematical and language processing was increased in both congenital and adult-onset blindness, but was stronger in the congenital group. Taken together, these studies indicate that long-range connectivity between occipital and higher-order cortical regions can be altered in both congenital and adult-onset blindness, but such connectivity is generally stronger in congenital or early blindness.

However, an alternative view questions whether occipital responses in blindness are truly differentiated in a task-selective manner. Rather than reflecting specialization for distinct cognitive functions, the widespread occipital activations observed across tasks may instead index sensitivity to general cognitive demands. Using hard-versus-easy contrasts across multiple tasks within the same blind participants, Duymuş et al. (2024) found robust and spatially widespread difficulty effects across bilateral occipital cortex. Moreover, occipital voxels showing the strongest difficulty effect in one task also showed similar effects in the other tasks, suggesting that their responses were not tied to a single task domain but instead reflected cross-task sensitivity to general demands. Importantly, these occipital regions also showed strong resting-state functional connectivity with the canonical frontoparietal multiple-demand network. On this basis, the authors proposed that the occipital cortex in blind individuals may be broadly incorporated into an MD-like functional system (Duymuş et al., 2024). This interpretation also points to a potential limitation of earlier studies. On the one hand, many studies supporting task selectivity in the occipital cortex likewise relied on contrasts between task conditions, yet such contrasts often capture not only differences in task domain itself, but also concurrent variation in cognitive load, response conflict, or retrieval demands. On the other hand, the hard-versus-easy contrasts used by Duymuş et al. (2024) were specifically designed to identify regions sensitive to cognitive load, and therefore cannot determine whether those same regions are also differentiated by task content. In other words, even if the same occipital voxels exhibit similar difficulty effects across multiple tasks, this does not rule out the possibility that they support different forms of information processing in different task contexts. In this sense, the two lines of evidence may be limited in opposite directions: the former may overattribute occipital responses to task domain, whereas the latter may under detect task-specific functional differentiation. This problem also extends to the functional connectivity findings in these studies, because many of them used ROI-based approaches in which occipital seed regions were defined on the basis of task contrasts. As a result, any mixture of task-category and difficulty-related effects embedded in the original contrast is likely to be carried forward into the connectivity analysis.

Beyond group-average effects, several studies have begun to examine interindividual variability in occipital functional connectivity in blind individuals, as well as the temporal stability of such variability. Sen et al. (2022) systematically compared variability in V1 functional connectivity between congenitally blind and sighted individuals. They found that, in the congenitally blind group, functional connectivity between V1 and frontal, parietal, and other visual regions showed substantially greater interindividual variability. Importantly, this increased variability was concentrated in connectivities that also showed systematic group-level differences, such as V1–frontal connectivity, which was on average stronger in blind individuals than in sighted controls but also markedly more variable across individuals. In addition, the strength of V1 connectivity with the left frontal cortex was associated with years of education, suggesting that postnatal experience may play a major role in shaping long-range occipital connectivity. On this basis, the authors argued that congenital blindness does not reorganize primary visual cortex along a single common trajectory, but instead increases interindividual variability in its functional connectivity; conversely, visual experience may normally serve to constrain and canalize these connectivity patterns (Sen et al., 2022). At the longitudinal level, Amaral et al. (2024) further examined the temporal stability of such individual differences. They found that, in congenitally blind individuals, V1 functional connectivity patterns remained highly stable over an interval of approximately 2 years. Individual identity was the primary factor accounting for connectivity differences, exceeding the contribution of scan condition (task vs. rest) and time point (Amaral et al., 2024). This evidence suggests that life experiences may contribute to systematic heterogeneity within the blind population. If such experiential heterogeneity directly shapes occipital functional reorganization or connectivity, it should be explicitly considered when examining occipital function and connectivity in blindness. One possibility, for example, is that within pre-existing anatomical constraints, congenital blindness may allow individual experiential histories to shape distinct patterns of network embedding.

A final methodological consideration concerns scan state and acquisition context. Several studies suggest that occipital functional connectivity estimates may differ between resting-state and task-state conditions, and between eyes-open and eyes-closed resting-state acquisitions (Guerreiro et al., 2021; Klinge et al., 2010; Pelland et al., 2017). These effects appear most clearly in connectivity between visual cortex and auditory or somatosensory cortices. Thus, scan state should be considered when comparing functional connectivity findings across studies. At present, however, this evidence remains limited in scope and does not directly explain the altered embedding of occipital cortex within language, frontoparietal control, or other higher-order cognitive networks in blindness.

Overall, current evidence on long-range functional connectivity of the occipital cortex in congenital blindness is compatible with both theoretical accounts. One set of findings is more consistent with the modality-independent, task-specific framework. In some higher-order visual areas, blind and sighted individuals show substantial similarity in long-range connectivity patterns, and these connectivity profiles can predict functional selectivity. This pattern suggests that the functional role of a cortical region remains strongly constrained by its pre-existing connectivity architecture. At the same time, a different set of findings shows selectively enhanced coupling between occipital cortex and several higher-order cognitive systems, including language, mathematics, executive control, and memory-related networks. These results indicate that, in the absence of visual input, visual cortex can become integrated into multiple higher-order cognitive systems, in line with the cognitively pluripotent account. Recent reviews, however, have argued that apparent preservation of functional and connectivity profiles in ventral visual cortex should be interpreted with caution. Saccone et al. (2024) noted that similarity in functional category selectivity and long-range connectivity profile is not sufficient to show that the same perceptual computations are implemented in higher-order visual areas in blind and sighted individuals. Even when a given region shows similar selectivity for the same stimulus category, or similar connectivity with other brain regions, this does not by itself establish that it represents or processes the same type of information (Saccone et al., 2024). The visual word form area provides a useful example. In sighted individuals, this region is typically involved in processing the visual form of written words. In blind individuals, by contrast, it may instead support higher-level speech-related processing, such as phonological or semantic processing (Kim et al., 2017). Under such circumstances, even if both groups show stronger connectivity between this region and the language network, the underlying processing content may still differ (Tian et al., 2023). Thus, preserved functional and connectivity profiles in higher-order visual cortex and increased integration into higher-order cognitive networks should not be treated as mutually exclusive interpretations.

## 5 Structural alterations in white matter connectivity

Functional connectivity provides one view of how the occipital cortex interacts with distributed brain systems in blindness, but these interactions are embedded within an underlying white matter architecture. Structural connectivity studies offer a complementary perspective by examining how visual deprivation affects the pathways linking occipital cortex with subcortical structures and other cortical systems. Existing evidence has mainly addressed three levels of organization: the visual afferent pathways and occipital white matter, interhemispheric and long-range association fibers involving occipital cortex, and, in a smaller number of studies, whole-brain structural network topology. The following section reviews these findings to clarify what is currently known about the structural connectivity profile of the blind occipital cortex.

Structural evidence in congenital and early blindness is most extensive for the visual afferent pathway and occipital white matter. The visual afferent pathway comprises the optic nerve, optic chiasm, optic tract, lateral geniculate nucleus (LGN), and optic radiations projecting to primary visual cortex. Evidence from voxel-based morphometry (VBM) and diffusion tensor imaging (DTI) converges in showing that, although the visual afferent pathway remains anatomically recognizable in congenitally or early blind individuals, the optic radiations and adjacent white matter show reduced volume and altered diffusion properties (Anurova et al., 2019; Noppeney, 2007; Ptito et al., 2008, 2021; Shimony et al., 2006; Shu et al., 2009a; Wang et al., 2013; Yu et al., 2007). More specifically, studies have reported reduced fractional anisotropy (FA) in the bilateral geniculocalcarine pathways of congenitally blind individuals, accompanied by increased radial diffusivity (RD) and mean diffusivity (MD), with no significant change in axial diffusivity (AD) (Shimony et al., 2006; Shu et al., 2009a; Yu et al., 2007). Reduced FA in the bilateral optic radiations has likewise been reported in congenital blindness (Wang et al., 2013), whereas volumetric reductions have been observed in the optic radiations, optic tracts, and lateral geniculate nucleus (Noppeney et al., 2005; Ptito et al., 2008, 2021). These alterations are not restricted to the afferent visual pathways, but extend into the occipital lobe, where reductions in both white matter volume and FA have also been reported (Anurova et al., 2019; Lao et al., 2015; Noppeney et al., 2005; Reislev et al., 2016a; Shimony et al., 2006). In diffusion imaging, lower FA is commonly interpreted as reflecting reduced fiber coherence or tissue organization, whereas higher RD and MD are often taken to indicate broader microstructural alterations, such as reduced myelination, axonal integrity, or tissue density (Beaulieu, 2002; Song et al., 2005; Zhang et al., 2022).

Whether these structural changes depend on the timing of visual loss has been examined through direct comparisons of congenital and late-onset blindness and through disorders involving progressive visual loss in adulthood. Wang et al. (2013) found that FA reductions in congenital blindness were concentrated in the bilateral optic radiations, whereas late-onset blindness was associated with a broader distribution of FA reductions involving the optic radiations, corpus callosum, and anterior thalamic radiation (Wang et al., 2013). However, Ptito et al. (2021) found comparable volume reductions in the optic nerves, optic chiasm, optic tracts, and lateral geniculate nucleus in congenital and late-onset blindness(Ptito et al., 2021). Collectively, reductions in white matter volume and altered diffusion properties in the visual afferent pathways and occipital white matter have generally been interpreted as reflecting multiple consequences of visual deprivation, including altered maturation, deafferentation, and secondary degeneration. Support for this interpretation comes from studies of retinitis pigmentosa (RP), which provides a model of progressive visual deprivation. Hofstetter et al. (2019) showed that FA in the optic tract and optic radiation was lower in patients with residual central tunnel vision than in normally sighted individuals, and was further reduced in completely blind patients, whereas RD showed the opposite pattern. These findings suggest that microstructural changes in the visual afferent pathway follow a graded degenerative pattern that tracks the severity of visual deprivation (Hofstetter et al., 2019). POAG provides a complementary model of partial and spatially heterogeneous visual-field loss. Consistent with previous evidence of white-matter alterations in the optic tracts and optic radiations following visual loss, Haykal et al. (2022) used neurite orientation dispersion and density imaging (NODDI) to further characterize visual-pathway microstructure in POAG. They reported a lower neurite density index (NDI) and a higher orientation dispersion index (ODI) in the optic tracts, together with a higher ODI in the optic radiations. These findings were interpreted as reduced neurite density and axonal coherence in the optic tracts and reduced axonal coherence in the optic radiations. Optic-tract NDI was also associated with visual-field severity.

At the level of thalamocortical connectivity, current evidence points to relative preservation of the macroscale projection pattern, alongside more localized changes in connectivity topography. Using probabilistic tractography based on diffusion-weighted imaging, Reislev et al. (2017) found no significant differences between congenitally blind and sighted individuals in the overall pattern or strength of thalamocortical connectivity, suggesting that the large-scale organization of thalamic projections to different cortical regions is largely preserved. At the same time, microstructural changes were observed in congenital blindness, including reduced thalamic volume, lower FA within the thalamus, and reduced FA in thalamo-occipital projection pathways (Reislev et al., 2017). A later voxel-wise study further suggested that this preservation at the macroscale may coexist with more subtle shifts in thalamic connectivity profiles: in congenitally blind individuals, occipital-connected thalamic regions showed reduced volume and connection probability, whereas temporal-connected regions showed increases in both measures, suggesting that the relative distribution of thalamic connectivity across cortical targets may be altered in congenital blindness (Marins et al., 2023). Overall, structural alterations in the visual afferent pathway show a high degree of consistency across studies. Blind individuals consistently show microstructural changes in this pathway, including reduced FA, increased RD, and volume loss, a pattern that is broadly consistent with classical deafferentation and secondary degeneration. These findings have been taken to indicate that the absence of visual input has a direct impact on white matter development within the afferent visual system.

Resting-state fMRI studies have reported reduced functional connectivity between interhemispheric homotopic occipital regions in blind individuals (Abboud and Cohen, 2019; Hou et al., 2017; Qin et al., 2013; Watkins et al., 2012). Consistent with these functional findings, structural studies have reported alterations in the splenium of the corpus callosum, the major commissural pathway linking homotopic occipital regions. In congenitally or early blind individuals, the splenium shows reduced volume and lower FA, with these changes mainly confined to posterior visual callosal sectors rather than distributed broadly across the corpus callosum (Ptito et al., 2008; Wang et al., 2013; Yu et al., 2007). A tractography-based quantitative analysis by Cavaliere et al. (2020) also reported reduced anatomical connectivity in the splenium and adjacent midbody of the corpus callosum, reflected in a lower number of white matter voxels structurally connected through these callosal regions. However, the same study also found a larger anterior commissure and increased streamline counts in its posterior branch in congenitally blind individuals. These findings suggest that commissural alterations in congenital blindness are not limited to a simple reduction in posterior callosal connectivity, but may involve a more complex pattern of plastic change across commissural pathways. Notably, in this study, resting-state connectivity between cortical regions linked by these commissural subdivisions did not differ reliably between groups (Cavaliere et al., 2020). These findings do not support a simple one-to-one correspondence between structural alterations in commissural pathways and reduced interhemispheric occipital functional connectivity.

Structural studies have also examined long-range association fibers linking the occipital cortex with temporal, frontal, and parietal regions, most notably the inferior longitudinal fasciculus and inferior fronto-occipital fasciculus of the ventral visual pathway, and the superior longitudinal fasciculus of the dorsal pathway. In congenital blindness, microstructural alterations are reported mainly in the posterior occipital portions of the inferior longitudinal fasciculus and inferior fronto-occipital fasciculus, where reduced FA is accompanied by increased RD, with no significant change in AD; under the same analysis, the superior longitudinal fasciculus does not show a comparable pattern (Reislev et al., 2016b).

In both congenital and late-onset blindness, microstructural alterations have been reported mainly in the posterior occipital portions of the inferior longitudinal fasciculus and inferior fronto-occipital fasciculus, where reduced FA is accompanied by increased RD, with no significant change in AD. Under the same analysis, the superior longitudinal fasciculus did not show a comparable pattern, and no significant differences were found between the congenital and late-onset blind groups (Reislev et al., 2016b). A similar pattern was reported in retinitis pigmentosa, where decreasing FA in the posterior inferior longitudinal fasciculus and inferior fronto-occipital fasciculus was accompanied by increasing RD as visual field coverage became progressively smaller (Hofstetter et al., 2019). These findings suggest that microstructural alterations in posterior ventral association fibers are not restricted to visual deprivation during early development.

Some studies have further attempted to relate the integrity of these association fibers to behavioral performance. FA in the right inferior longitudinal fasciculus was positively associated with auditory spatial working-memory accuracy in both early blind and sighted participants. At the same time, the association between occipital activity and tract FA differed in direction across groups, being positive in the blind but negative in the sighted(Anurova et al., 2019). Based on this pattern, the authors suggested that cross-modal auditory processing in occipital cortex may depend on changes in how an existing auditory–frontal–occipital structural network is functionally utilized. Notably, findings concerning the ventral visual pathway in blindness present an inconsistent picture across white matter microstructure, functional connectivity, and functional activation. At the functional and functional-connectivity levels, the dominant conclusion has been relative preservation: ventral occipitotemporal regions in blind individuals often retain functional selectivity and long-range connectivity profiles broadly similar to those observed in sighted individuals, although the relevant information reaches these regions through non-visual modalities. In contrast, structural studies of the same pathway have consistently reported reduced FA and increased RD in posterior ventral association fibers, a pattern usually interpreted as deafferentation or secondary degeneration following visual deprivation. Yet when these white matter measures are related to behavior or occipital activity, they are sometimes discussed in terms of compensatory plasticity or cross-modal reorganization. These different interpretations are difficult to reconcile.

Only a small number of studies have moved beyond individual tracts and examined the whole-brain structural connectome in blind individual. Graph-theoretical analyses of tractography-derived structural networks show that early blind individuals exhibit lower global efficiency and network density, along with increased characteristic path length (Shu et al., 2009b; Zhou et al., 2022). In graph-theoretical terms, characteristic path length refers to the average minimum number of network edges required to connect pairs of brain regions, rather than their physical distance. Lower global efficiency and longer path length therefore suggest that the structural network supports less efficient inter-regional communication at the whole-brain level. Regional topological alterations have also been reported. Specifically, degree centrality, which reflects the number of connections a region maintains within the network, was increased in the cerebellum, supplementary motor area, and fusiform gyrus, but decreased in frontal regions (Wen et al., 2022). On this basis, the authors interpreted these changes as indicating altered roles of specific regions within the overall network architecture. Connectome-level analyses thus point to broad alterations in large-scale structural topology, most consistently reflected in reduced network efficiency and increased path length. These findings describe how whole-brain white matter networks are reorganized in blindness, but they remain largely descriptive. On their own, however, these measures do not explain the functional recruitment of occipital cortex in blindness, or specify how structural network alterations are aligned with functional connectivity changes.

Evidence directly linking structural connectivity to functional organization in occipital cortex remains limited. Wang et al. (2017) found that, in both congenitally blind and sighted individuals, the structural connectivity profile of the parahippocampal cortex significantly predicted its functional selectivity for large, man-made objects. Moreover, models trained in one group generalized to the other with comparable accuracy, suggesting that the mapping between connectivity profile and functional organization in this region is preserved in the absence of visual experience (Wang et al., 2017).

A final key issue concerns the relationship between white-matter structural connectivity and functional connectivity. To address this issue, several studies have compared structural and functional connectivity changes within the same participants. For example, Bauer et al. (2017) found a relatively high correspondence between white-matter structural connectivity and resting-state functional connectivity within both early blind and sighted groups. However, when comparing group differences, changes in structural connectivity did not significantly correlate with changes in functional connectivity (Bauer et al., 2017). A similar lack of one-to-one correspondence has also been noted in more specific pathway-based analyses, including commissural pathways linking the two hemispheres (Cavaliere et al., 2020). More recently, this issue has also been addressed in terms of structural–functional coupling (SC–FC coupling), which indexes how closely functional connectivity follows the underlying structural connectome. Stronger coupling reflects greater alignment between functional interactions and anatomical pathways, whereas weaker coupling reflects a looser structure–function relationship (Fotiadis et al., 2024). Only one study has directly investigated SC–FC coupling in blindness. At the whole-brain level, SC–FC coupling did not differ significantly between congenitally blind and sighted individuals. Reduced coupling was observed within intrahemispheric networks in the congenitally blind group. In addition, earlier Braille onset and better Braille reading performance were associated with weaker SC–FC decoupling (Jiao et al., 2025). The authors interpreted this pattern as indicating that Braille reading mitigated SC–FC decoupling, suggesting a recovery toward a more typical state of structure–function coupling. This interpretation is plausible, but it should be treated with caution. In the broader SC-FC literature, stronger coupling is not uniformly equivalent to a more typical or more adaptive state; coupling strength varies with cortical hierarchy, development, and cognitive context, and weaker coupling may also reflect greater functional flexibility (Baum et al., 2020; Fotiadis et al., 2024). In congenital blindness, reduced intrahemispheric SC–FC coupling therefore does not necessarily indicate damage to an already established structure–function relationship. It may instead reflect a different developmental organization in which functional connectivity is reorganized relative to the underlying structural connectome. Accordingly, the association between Braille experience and stronger SC–FC coupling suggests that tactile reading experience may help maintain or reshape the alignment between structural and functional networks under visual deprivation, but it does not by itself establish a recovery toward a normal structure–function relationship.

## 6 Discussion and open questions

The findings reviewed above suggest that connectivity in the blind occipital cortex reflects neither simple preservation nor unrestricted reorganization. Rather, they point to a constrained form of plasticity, in which retained organizational features and altered developmental experience jointly shape the functional organization of occipital cortex. The central challenge, therefore, is to explain how these two aspects coexist across occipital regions and give rise to different patterns of connectivity and function. In this sense, the emerging picture is a mosaic in which different occipital circuits retain different pre-existing biases, while sensory history and repeated task engagement differentially modify their contribution to non-visual and higher-order cognitive processing.

A more specific issue concerns whether the coexistence of preservation and reorganization can be understood in terms of regional variation across occipital and occipitotemporal cortex. A natural extension of this idea is to map this variation onto the distinction between early and higher-order visual cortex, with more anterior higher-order regions showing greater similarity to the sighted pattern and more posterior early visual regions showing greater divergence (Wang et al., 2015). This finding points to a meaningful spatial gradient in connectivity similarity between blind and sighted individuals. However, other evidence is not fully consistent with a simple hierarchical interpretation. In early visual cortex, preserved retinotopy-related intrinsic organization indicates that these regions do not simply lose their original organizational principles in the absence of visual input. Conversely, higher-order occipital and occipitotemporal regions may retain sighted-like long-range connectivity profiles while also showing altered embedding within higher-order cognitive networks. Thus, preservation and reorganization do not appear to map cleanly onto the distinction between “early” and “higher-order” cortex. One possibility is that the outcome of visual deprivation reflects a mosaic of circuits with different pre-existing connectivity biases and different susceptibility to experience-dependent modification. In addition, for early visual cortex, existing work has primarily examined whether local or intra-occipital functional connectivity preserves retinotopy-related organization, whereas the whole-brain connectivity profile of early visual cortex, its variability across blind individuals, and its relationship to task-based functional recruitment remain less systematically characterized.

The coexistence of preserved and altered connectivity can also be considered in relation to the account proposed by Makin and Krakauer (2023). Their framework emphasizes that substantial functional changes may emerge through the differential potentiation of pre-existing inputs and circuits, without requiring a cortical region to acquire an entirely new anatomical architecture. This interpretation is compatible with several findings reviewed here, including the preservation of retinotopy-related intrinsic organization, the broadly similar long-range connectivity profiles of some ventral occipitotemporal regions in blind and sighted individuals, and the alteration of functional interactions in the absence of correspondingly extensive structural changes. More generally, pre-existing architecture and experience-dependent reorganization are not mutually exclusive explanations. As emphasized by Makin and Krakauer (2023), changes in cortical activation and connectivity do not necessarily indicate the establishment of an entirely new structural or functional organization; they may instead reflect the strengthening or reweighting of pre-existing network architecture. We agree with this aspect of their account. Tian et al. (2026), for example, found that the enhanced functional connectivity between occipital and prefrontal regions observed in congenitally blind adults appears to build on a connectivity pattern already present in infancy. In infants, secondary visual cortex showed stronger functional connectivity with prefrontal cortex than with non-visual sensory–motor cortices, resembling the connectivity profile observed in congenitally blind adults. These findings suggest that the increased occipital–prefrontal functional connectivity associated with higher-order cognitive processing in blindness may develop through the strengthening and further differentiation of an early connectivity scaffold, rather than through the formation of an entirely new pathway. However, the early presence of this connectivity scaffold does not preclude the occipital cortex from acquiring a different computational or functional role during development. Rather, pre-existing occipital–prefrontal connectivity may provide a route through which higher-order information reaches the occipital cortex in the absence of visual input, thereby supporting its involvement in language, mathematics, executive control, and other higher-order cognitive functions. Future studies should combine task-based fMRI with connectivity analyses to clarify whether occipital activity reflects specific computations involved in higher-order cognitive function. From a connectivity perspective, a key question is how the whole-brain connectivity fingerprints of these occipital regions are altered, including changes in their relative connectivity with multiple systems, their position within large-scale network organization, and their affiliation with higher-order cognitive networks. Existing resting-state findings of enhanced connectivity with selected frontal, parietal, or language-related regions are suggestive, but do not yet establish such large-scale network integration. Combine with task-dependent connectivity analyses could further test how these altered connectivity profiles are expressed during cognition as selective interactions with the relevant higher-order cognitive networks, and whether these interactions correspond to the information represented in occipital cortex. Finally, the functional relevance of these changes could be assessed by testing whether occipital representations and connectivity patterns predict behavioral performance across individuals.

A second issue concerns how structural connectivity should be interpreted in relation to functional connectivity and occipital recruitment in blindness. Compared with functional connectivity research, structural connectivity studies in blindness have more often been organized around a deficit-oriented perspective. Whether examining individual white matter tracts or whole-brain structural networks, most studies still take as their starting point the question of which pathways show reduced volume or altered microstructural integrity, and whether the structural connectome shows reduced efficiency, lower density, or increased path length in blind individuals. This framework may be useful for characterizing degenerative changes along the visual afferent system, but it is less informative for explaining how altered structural connectivity relates to the functional reorganization of the occipital cortex in blindness. A further consideration is that functional reorganization does not necessarily require the formation of new large-scale white matter pathways. To date, there is little evidence that blindness leads to entirely new long-range anatomical tracts. Instead, animal studies suggest that sensory deprivation can modify the efficacy and relative weighting of existing cortical and subcortical circuits. In mice, short-term visual deprivation strengthens thalamocortical inputs to the spared auditory cortex and improves auditory cortical encoding (Petrus et al., 2014). It also shifts the balance between feedforward and intracortical inputs in opposite directions across spared and deprived sensory cortices, strengthening feedforward processing in auditory cortex while strengthening lateral intracortical inputs in visual cortex (Petrus et al., 2015). In addition, visual deprivation refines excitatory and inhibitory intracortical input patterns in auditory cortex without changing the overall excitation–inhibition balance, suggesting that functional changes may also arise from altered input selectivity and circuit reliability rather than from gross anatomical rewiring (Meng et al., 2015). Although these findings come primarily from animal models and often concern short-term deprivation in juveniles or adults, they illustrate a mechanism by which functional interactions may be modified within pre-existing anatomical pathways. At the same time, human structural imaging studies do indicate that blindness is associated with changes in several white matter pathways beyond the primary visual afferent system. As discussed in the previous section, blindness is associated with alterations in several white matter pathways, including posterior commissural pathways linking occipital regions across hemispheres and posterior portions of ventral association fibers such as the inferior longitudinal fasciculus and inferior fronto-occipital fasciculus. The key issue is therefore not only whether structural pathways are preserved or reduced, but how existing anatomical architecture, altered white-matter properties, and experience-dependent changes in functional coupling jointly shape the network embedding of the blind occipital cortex.

Another perspective on the structure–function relationship is provided by structure–function coupling, which quantifies the alignment between structural and functional connectivity and allows this relationship to be examined across the cortical hierarchy. Evidence in blindness, however, remains limited. Existing findings have mainly addressed whole-brain or intrahemispheric coupling (Jiao et al., 2025), leaving open how SC–FC coupling varies across the occipital cortex itself. Importantly, studies in the general population have shown that SC–FC coupling varies systematically across the cortical hierarchy, with stronger coupling in primary sensory regions and weaker coupling in higher-order association cortices. This raises the possibility that future studies could examine whether SC–FC coupling is also regionally differentiated within the blind occipital cortex, and whether such differentiation tracks the balance between preserved connectivity constraints and experience-dependent functional reorganization.

A fourth issue concerns how to disentangle the effects of visual experience from those of blindness-related plasticity. Differences between blind and sighted adults are often attributed to reorganization following visual deprivation. However, this interpretation assumes that the sighted adult pattern represents a neutral baseline, rather than an outcome shaped by visual experience. Accumulating evidence suggests that visual experience plays an important role in shaping the organization of occipital connectivity (Crair, 1999; Roy et al., 2020; Stellwagen and Shatz, 2002). In sighted individuals, shared visual input is associated with more consistent connectivity patterns across individuals, whereas the absence of such shared experience in blindness is linked to substantially greater inter-individual variability in occipital functional connectivity (Sen et al., 2022). Developmental comparisons provide a more direct way to distinguish these possibilities. Connectivity differences commonly observed between blind and sighted adults include stronger occipital–prefrontal functional connectivity and weaker connectivity between occipital cortex and non-visual sensory–motor systems in blindness (Butt et al., 2013; Heine et al., 2015; Liu et al., 2007; Qin et al., 2013; Striem-Amit et al., 2015; Yu et al., 2008). These patterns are typically interpreted as reflecting plastic reorganization following visual deprivation, whereby the loss of visual input reduces multisensory integration with auditory and somatosensory systems, while increased recruitment of higher-order cognitive functions enhances occipital–prefrontal coupling. Tian et al. (2026) showed that these adult group differences are better understood in relation to developmental change. The long-range connectivity profile of neonatal occipital cortex was more similar to that of congenitally blind adults than to that of sighted adults. This comparison indicates that visual experience contributes actively to the postnatal transformation of occipital connectivity in the sighted brain. It therefore suggests that visual experience appears to play an active role in shaping the sighted adult connectivity profile after birth. Thus, differences between blind and sighted adults may arise not only from plastic changes following blindness, but also from experience-dependent modification of connectivity in the sighted brain. This developmental perspective shifts the focus from static group differences to the trajectories through which connectivity patterns emerge. Most existing studies have focused on blind adults, with relatively little work examining blind children or longitudinal change. It therefore remains unclear when blind and sighted trajectories begin to diverge, which connectivity features remain stable across development, and which are progressively shaped by visual, tactile, auditory, linguistic, and navigational experience. A key question is not simply whether occipital connectivity differs between blind and sighted adults, but how these patterns develop from infancy through childhood under different sensory and behavioral conditions.

A related developmental question is whether age at blindness onset constrains the form of occipital plasticity. Studies of occipital recruitment for higher-order cognition show a relatively consistent pattern, with weaker or less differentiated responses following adult-onset blindness than following congenital or early blindness (Bedny et al., 2012; Kanjlia et al., 2019; Pant et al., 2020). In contrast, findings for other forms of plasticity, including local occipital organization, long-range functional connectivity, and white-matter structure, are less consistent. Some changes are shared across congenital and later-onset blindness, whereas others differ in strength, spatial extent, or the specific connectivity property affected. This variability may partly reflect the broad onset ranges used in some studies, which combine individuals whose blindness began in childhood and adulthood within a single later-onset group. It may also indicate that different forms of plasticity are constrained by different sensitive periods. Future studies should characterize onset age more precisely and distinguish it from blindness duration to determine whether different aspects of occipital organization follow distinct developmental time courses.

Taken together, these issues point to a broader limitation in the current literature. Existing studies have often relied on coarse-grained group comparisons, predefined regional frameworks, or separate analyses of structural and functional connectivity. As a result, the field still lacks an integrated picture of how the blind occipital cortex is structurally connected, functionally embedded, and developmentally shaped. It also remains unclear how this connectivity profile emerges across development, and how it is influenced by visual deprivation, visual experience, and blindness-related experiences such as tactile literacy, non-visual spatial navigation, mobility training, assistive-technology use, and intensive reliance on auditory or haptic information. Future work should therefore move beyond static adult group comparisons and predefined visual-cortex frameworks, and ask how occipital connectivity and function are configured in blindness at the level of individual topographies, connectivity gradients, and network embedding. Developmental, longitudinal, and individual-level approaches will be especially important for determining how preserved connectivity constraints and experience-dependent reorganization jointly give rise to the functional profile of the blind occipital cortex.

## 7 Conclusion

In summary, the available evidence indicates that the organization of the occipital cortex in blindness cannot be fully captured by either a simple preservation account or a purely reorganization-based account. Instead, occipital regions exhibit a combination of preserved intrinsic organization and altered large-scale network embedding, consistent with a mosaic in which circuits with different pre-existing connectivity profiles undergo different patterns of experience-dependent modification. This view highlights the importance of connectivity-based approaches for determining how pre-existing constraints and developmental experience jointly shape cortical organization in the absence of visual input.

## References

[ref1] Abboud S. Cohen L. (2019). Distinctive interaction between cognitive networks and the visual cortex in early blind individuals. Cereb. Cortex 29, 4725–4742. doi: 10.1093/cercor/bhz006, 30715236

[ref2] Amalric M. Denghien I. Dehaene S. (2018). On the role of visual experience in mathematical development: evidence from blind mathematicians. Dev. Cogn. Neurosci. 30, 314–323. doi: 10.1016/j.dcn.2017.09.007, 29033221 PMC5833949

[ref3] Amaral L. Thomas P. Amedi A. Striem-Amit E. (2024). Longitudinal stability of individual brain plasticity patterns in blindness. Proc. Natl. Acad. Sci. 121:e2320251121. doi: 10.1073/pnas.2320251121, 39078671 PMC11317565

[ref4] Amedi A. Floel A. Knecht S. Zohary E. Cohen L. G. (2004). Transcranial magnetic stimulation of the occipital pole interferes with verbal processing in blind subjects. Nat. Neurosci. 7, 1266–1270. doi: 10.1038/nn1328, 15467719

[ref5] Amedi A. Hofstetter S. Maidenbaum S. Heimler B. (2017). Task selectivity as a comprehensive principle for brain organization. Trends Cogn. Sci. 21, 307–310. doi: 10.1016/j.tics.2017.03.007, 28385460

[ref6] Anurova I. Carlson S. Rauschecker J. P. (2019). Overlapping anatomical networks convey Cross-modal suppression in the sighted and Coactivation of “visual” and auditory cortex in the blind. Cereb. Cortex 29, 4863–4876. doi: 10.1093/cercor/bhz021, 30843062 PMC7112717

[ref7] Arbel R. Heimler B. Amedi A. (2022). Face shape processing via visual-to-auditory sensory substitution activates regions within the face processing networks in the absence of visual experience. Front. Neurosci. 16:921321. doi: 10.3389/fnins.2022.921321, 36263367 PMC9576157

[ref8] Barttfeld P. Abboud S. Lagercrantz H. Adén U. Padilla N. Edwards A. D. . (2018). A lateral-to-mesial organization of human ventral visual cortex at birth. Brain Struct. Funct. 223, 3107–3119. doi: 10.1007/s00429-018-1676-3 29752588

[ref9] Bauer C. M. Hirsch G. V. Zajac L. Koo B. B. Collignon O. Merabet L. B. (2017). Multimodal MR-imaging reveals large-scale structural and functional connectivity changes in profound early blindness. PLoS One 12:e0173064. doi: 10.1371/journal.pone.0173064, 28328939 PMC5362049

[ref10] Baum G. L. Cui Z. Roalf D. R. Ciric R. Betzel R. F. Larsen B. . (2020). Development of structure–function coupling in human brain networks during youth. Proc. Natl. Acad. Sci. USA 117, 771–778. doi: 10.1073/pnas.1912034117, 31874926 PMC6955327

[ref11] Beaulieu C. (2002). The basis of anisotropic water diffusion in the nervous system – a technical review. NMR Biomed. 15, 435–455. doi: 10.1002/nbm.782, 12489094

[ref12] Bedny M. (2017). Evidence from blindness for a cognitively pluripotent cortex. Trends Cogn. Sci. 21, 637–648. doi: 10.1016/j.tics.2017.06.003, 28821345

[ref13] Bedny M. Konkle T. Pelphrey K. Saxe R. Pascual-Leone A. (2010). Sensitive period for a multimodal response in human visual motion area MT/MST. Curr. Biol. 20, 1900–1906. doi: 10.1016/j.cub.2010.09.044, 20970337 PMC2998392

[ref14] Bedny M. Pascual-Leone A. Dodell-Feder D. Fedorenko E. Saxe R. (2011). Language processing in the occipital cortex of congenitally blind adults. Proc. Natl. Acad. Sci. USA 108, 4429–4434. doi: 10.1073/pnas.1014818108, 21368161 PMC3060248

[ref15] Bedny M. Pascual-Leone A. Dravida S. Saxe R. (2012). A sensitive period for language in the visual cortex: distinct patterns of plasticity in congenitally versus late blind adults. Brain Lang. 122, 162–170. doi: 10.1016/j.bandl.2011.10.005, 22154509 PMC3536016

[ref16] Biswal B. Zerrin Yetkin F. Haughton V. M. Hyde J. S. (1995). Functional connectivity in the motor cortex of resting human brain using echo-planar MRI. Magn. Reson. Med. 34, 537–541. doi: 10.1002/mrm.1910340409, 8524021

[ref17] Bock A. S. Binda P. Benson N. C. Bridge H. Watkins K. E. Fine I. (2015). Resting-state retinotopic organization in the absence of retinal input and visual experience. J. Neurosci. 35, 12366–12382. doi: 10.1523/JNEUROSCI.4715-14.2015, 26354906 PMC4563031

[ref18] Burton H. Diamond J. B. McDermott K. B. (2003). Dissociating cortical regions activated by semantic and phonological tasks: a fMRI study in blind and sighted people. J. Neurophysiol. 90, 1965–1982. doi: 10.1152/jn.00279.2003, 12789013 PMC3705560

[ref19] Burton H. Sinclair R. J. Agato A. (2012). Recognition memory for braille or spoken words: an fMRI study in early blind. Brain Res. 1438, 22–34. doi: 10.1016/j.brainres.2011.12.032, 22251836 PMC3273673

[ref20] Butt O. H. Benson N. C. Datta R. Aguirre G. K. (2013). The fine-scale functional correlation of striate cortex in sighted and blind people. J. Neurosci. 33, 16209–16219. doi: 10.1523/JNEUROSCI.0363-13.2013, 24107953 PMC3792460

[ref21] Butt O. H. Benson N. C. Datta R. Aguirre G. K. (2015). Hierarchical and homotopic correlations of spontaneous neural activity within the visual cortex of the sighted and blind. Front. Hum. Neurosci. 9, 1–11. doi: 10.3389/fnhum.2015.00025, 25713519 PMC4322716

[ref22] Carvalho J. Invernizzi A. Ahmadi K. Hoffmann M. B. Renken R. J. Cornelissen F. W. (2020). Micro-probing enables fine-grained mapping of neuronal populations using fMRI. NeuroImage 209:116423. doi: 10.1016/j.neuroimage.2019.116423, 31811903

[ref23] Carvalho J. Invernizzi A. Martins J. Renken R. J. Cornelissen F. W. (2022). Local neuroplasticity in adult glaucomatous visual cortex. Sci. Rep. 12:21981. doi: 10.1038/s41598-022-24709-1, 36539453 PMC9767937

[ref24] Cavaliere C. Aiello M. Soddu A. Laureys S. Reislev N. L. Ptito M. . (2020). Organization of the commissural fiber system in congenital and late-onset blindness. NeuroImage 25:102133. doi: 10.1016/j.nicl.2019.102133, 31945651 PMC6965724

[ref25] Collignon O. Dormal G. Albouy G. Vandewalle G. Voss P. Phillips C. . (2013). Impact of blindness onset on the functional organization and the connectivity of the occipital cortex. Brain 136, 2769–2783. doi: 10.1093/brain/awt176 23831614

[ref26] Collignon O. Vandewalle G. Voss P. Albouy G. Charbonneau G. Lassonde M. . (2011). Functional specialization for auditory-spatial processing in the occipital cortex of congenitally blind humans. Proc. Natl. Acad. Sci. 108, 4435–4440. doi: 10.1073/pnas.1013928108, 21368198 PMC3060256

[ref27] Crair M. C. (1999). Neuronal activity during development: permissive or instructive? Curr. Opin. Neurobiol. 9, 88–93. doi: 10.1016/S0959-4388(99)80011-7, 10072369

[ref28] Deen B. Saxe R. Bedny M. (2015). Occipital cortex of blind individuals is functionally coupled with executive control areas of frontal cortex. J. Cogn. Neurosci. 27, 1633–1647. doi: 10.1162/jocn_a_00807, 25803598

[ref29] Duymuş H. Verma M. Güçlütürk Y. Öztürk M. Varol A. B. Kurt Ş. . (2024). The visual cortex in the blind but not the auditory cortex in the deaf becomes multiple-demand regions. Brain 147, 3624–3637. doi: 10.1093/brain/awae187, 38864500 PMC11449128

[ref30] Felleman D. J. Van Essen D. C. (1991). Distributed hierarchical processing in the primate cerebral cortex. Cereb. Cortex 1, 1–47. doi: 10.1093/cercor/1.1.1 1822724

[ref31] Fotiadis P. Parkes L. Davis K. A. Satterthwaite T. D. Shinohara R. T. Bassett D. S. (2024). Structure–function coupling in macroscale human brain networks. Nat. Rev. Neurosci. 25, 688–704. doi: 10.1038/s41583-024-00846-6, 39103609

[ref32] Guerreiro M. J. S. Linke M. Lingareddy S. Kekunnaya T. acts of opening and closing the eyes are of importance for congenital blindness: E. from resting-state fMRI Röder B. (2021). The effect of congenital blindness on resting-state functional connectivity revisited. Sci. Rep. 11, 1–14. doi: 10.1038/s41598-021-91976-9 34127748 PMC8203782

[ref33] Haak K. V. Winawer J. Harvey B. M. Renken R. Dumoulin S. O. Wandell B. A. . (2013). Connective field modeling. NeuroImage 66, 376–384. doi: 10.1016/j.neuroimage.2012.10.037, 23110879 PMC3769486

[ref34] Haupt M. Graumann M. Teng S. Kaltenbach C. Cichy R. (2024). The transformation of sensory to perceptual braille letter representations in the visually deprived brain. eLife 13:RP98148. doi: 10.7554/eLife.98148, 39630852 PMC11616995

[ref35] Haykal S. Invernizzi A. Carvalho J. Jansonius N. M. Cornelissen F. W. (2022). Microstructural visual pathway white matter alterations in primary open-angle glaucoma: a neurite orientation dispersion and density imaging study. Am. J. Neuroradiol. 43, 756–763. doi: 10.3174/ajnr.A7495, 35450857 PMC9089264

[ref36] He C. Peelen M. V. Han Z. Lin N. Caramazza A. Bi Y. (2013). Selectivity for large nonmanipulable objects in scene-selective visual cortex does not require visual experience. NeuroImage 79, 1–9. doi: 10.1016/j.neuroimage.2013.04.051, 23624496

[ref37] Heimler B. Striem-Amit E. Amedi A. (2015). Origins of task-specific sensory-independent organization in the visual and auditory brain: neuroscience evidence, open questions and clinical implications. Curr. Opin. Neurobiol. 35, 169–177. doi: 10.1016/j.conb.2015.09.001, 26469211

[ref38] Heine L. Bahri M. A. Cavaliere C. Soddu A. Laureys S. Ptito M. . (2015). Prevalence of increases in functional connectivity in visual, somatosensory and language areas in congenital blindness. Front. Neuroanat. 9:86. doi: 10.3389/fnana.2015.00086, 26190978 PMC4486836

[ref39] Heinzle J. Kahnt T. Haynes J.-D. (2011). Topographically specific functional connectivity between visual field maps in the human brain. NeuroImage 56, 1426–1436. doi: 10.1016/j.neuroimage.2011.02.077, 21376818

[ref40] Hofstetter S. Sabbah N. Mohand-Saïd S. Sahel J.-A. Habas C. Safran A. B. . (2019). The development of white matter structural changes during the process of deterioration of the visual field. Sci. Rep. 9:2085. doi: 10.1038/s41598-018-38430-5, 30765782 PMC6375971

[ref41] Hölig C. Föcker J. Best A. Röder B. Büchel C. (2014). Crossmodal plasticity in the fusiform gyrus of late blind individuals during voice recognition. NeuroImage 103, 374–382. doi: 10.1016/j.neuroimage.2014.09.050, 25280451

[ref42] Hou F. Liu X. Zhou Z. Zhou J. Li H. (2017). Reduction of interhemispheric functional brain connectivity in early blindness: a resting-state fMRI study. Biomed. Res. Int. 2017, 1–8. doi: 10.1155/2017/6756927, 28656145 PMC5471583

[ref43] Innocenti G. M. Price D. J. (2005). Exuberance in the development of cortical networks. Nat. Rev. Neurosci. 6, 955–965. doi: 10.1038/nrn1790, 16288299

[ref44] Invernizzi A. Gravel N. Haak K. V. Renken R. J. Cornelissen F. W. (2021). Assessing uncertainty and reliability of connective field estimations from resting state fMRI activity at 3T. Front. Neurosci. 15:625309. doi: 10.3389/fnins.2021.625309, 33692669 PMC7937930

[ref9001] Invernizzi A. Carvalho J. C. Martins J. Jansonius N. M. Renken R. J. Cornelissen F. W. (2025). Primary open angle glaucoma is associated with cortico-cortical receptive fields changes in early visual cortex. medRxiv. doi: 10.1101/2025.01.03.25319969, 40622597

[ref45] Jiang F. Stecker G. C. Boynton G. M. Fine I. (2016). Early blindness results in developmental plasticity for auditory motion processing within auditory and occipital cortex. Front. Hum. Neurosci. 10:324. doi: 10.3389/fnhum.2016.00324, 27458357 PMC4932114

[ref46] Jiao S. Wang K. Zeng J. Cui Z. Luo Y. Han Z. (2025). “Touching” the brain: braille reading mitigates the SC–FC decoupling of brain networks in congenital blindness. Brain Struct. Funct. 230:114. doi: 10.1007/s00429-025-02975-9, 40622597

[ref47] Kanjlia S. Lane C. Feigenson L. Bedny M. (2016). Absence of visual experience modifies the neural basis of numerical thinking. Proc. Natl. Acad. Sci. USA 113, 11172–11177. doi: 10.1073/pnas.1524982113, 27638209 PMC5056030

[ref48] Kanjlia S. Loiotile R. E. Harhen N. Bedny M. (2021). ‘Visual’ cortices of congenitally blind adults are sensitive to response selection demands in a go/no-go task. NeuroImage 236:118023. doi: 10.1016/j.neuroimage.2021.118023, 33862241 PMC8249356

[ref49] Kanjlia S. Pant R. Bedny M. (2019). Sensitive period for cognitive repurposing of human visual cortex. Cereb. Cortex 29, 3993–4005. doi: 10.1093/cercor/bhy280, 30418533 PMC6686750

[ref50] Kim J. S. Kanjlia S. Merabet L. B. Bedny M. (2017). Development of the visual word form area requires visual experience: evidence from blind braille readers. J. Neurosci. Off. J. Soc. Neurosci. 37, 11495–11504. doi: 10.1523/JNEUROSCI.0997-17.2017, 29061700 PMC5700429

[ref51] Klinge C. Eippert F. Röder B. Büchel C. (2010). Corticocortical connections mediate primary visual cortex responses to auditory stimulation in the blind. J. Neurosci. Off. J. Soc. Neurosci. 30, 12798–12805. doi: 10.1523/JNEUROSCI.2384-10.2010, 20861384 PMC6633575

[ref52] Lane C. Kanjlia S. Omaki A. Bedny M. (2015). “Visual” cortex of congenitally blind adults responds to syntactic movement. J. Neurosci. 35, 12859–12868. doi: 10.1523/JNEUROSCI.1256-15.2015, 26377472 PMC6795209

[ref53] Lao Y. Kang Y. Collignon O. Brun C. Kheibai S. B. Alary F. . (2015). A study of brain white matter plasticity in early blinds using tract-based spatial statistics and tract statistical analysis. Neuroreport 26, 1151–1154. doi: 10.1097/WNR.0000000000000488, 26559727

[ref54] Li J. Osher D. E. Hansen H. A. Saygin Z. M. (2020). Innate connectivity patterns drive the development of the visual word form area. Sci. Rep. 10:18039. doi: 10.1038/s41598-020-75015-7, 33093478 PMC7582172

[ref55] Liu Y. Yu C. Liang M. Li J. Tian L. Zhou Y. . (2007). Whole brain functional connectivity in the early blind. Brain 130, 2085–2096. doi: 10.1093/brain/awm121, 17533167

[ref56] Lyon D. C. Kaas J. H. (2002). Evidence from V1 connections for both dorsal and ventral subdivisions of V3 in three species of new world monkeys. J. Comp. Neurol. 449, 281–297. doi: 10.1002/cne.10297, 12115680

[ref57] Makin T. R. Krakauer J. W. (2023). Against cortical reorganisation. eLife 12:e84716. doi: 10.7554/eLife.84716, 37986628 PMC10662956

[ref58] Marins T. F. Russo M. Rodrigues E. C. Monteiro M. Moll J. Felix D. . (2023). Reorganization of thalamocortical connections in congenitally blind humans. Hum. Brain Mapp. 44, 2039–2049. doi: 10.1002/hbm.26192, 36661404 PMC9980890

[ref59] Mattioni S. Rezk M. Battal C. Bottini R. Mendoza K. E. C. Oosterhof N. N. . (2020). Categorical representation from sound and sight in the ventral occipito-temporal cortex of sighted and blind. eLife 9, 1–33. doi: 10.7554/eLife.50732, 32108572 PMC7108866

[ref60] Mattioni S. Rezk M. Battal C. Vadlamudi J. Collignon O. (2022). Impact of blindness onset on the representation of sound categories in occipital and temporal cortices. eLife 11:e79370. doi: 10.7554/eLife.79370, 36070354 PMC9451537

[ref61] Matuszewski J. Bola Ł. Collignon O. Marchewka A. (2025). Similar computational hierarchies for Reading and speech in the occipital cortex of sighed and blind: converging evidence from fMRI and chronometric TMS. J. Neurosci. 45:e1153242024. doi: 10.1523/JNEUROSCI.1153-24.2024, 40032525 PMC12079739

[ref62] Meng X. Kao J. P. Y. Lee H.-K. Kanold P. O. (2015). Visual deprivation causes refinement of intracortical circuits in the auditory cortex. Cell Rep. 12, 955–964. doi: 10.1016/j.celrep.2015.07.018, 26235625 PMC4719125

[ref63] Merabet L. Thut G. Murray B. Andrews J. Hsiao S. Pascual-Leone A. (2004). Feeling by sight or seeing by touch? Neuron 42, 173–179. doi: 10.1016/S0896-6273(04)00147-3, 15066274

[ref64] Musz E. Loiotile R. Chen J. Cusack R. Bedny M. (2022). Naturalistic stimuli reveal a sensitive period in cross modal responses of visual cortex: evidence from adult-onset blindness. Neuropsychologia 172:108277. doi: 10.1016/j.neuropsychologia.2022.108277, 35636634 PMC9648859

[ref65] Noppeney U. (2007). The effects of visual deprivation on functional and structural organization of the human brain. Neurosci. Biobehav. Rev. 31, 1169–1180. doi: 10.1016/j.neubiorev.2007.04.012, 17597210

[ref66] Noppeney U. Friston K. J. Ashburner J. Frackowiak R. Price C. J. (2005). Early visual deprivation induces structural plasticity in gray and white matter. Curr. Biol. 15, R488–R490. doi: 10.1016/j.cub.2005.06.053, 16005276

[ref67] Pant R. Kanjlia S. Bedny M. (2020). A sensitive period in the neural phenotype of language in blind individuals. Dev. Cogn. Neurosci. 41:100744. doi: 10.1016/j.dcn.2019.100744, 31999565 PMC6994632

[ref68] Pascual-Leone A. Amedi A. Fregni F. Merabet L. B. (2005). The plastic human brain cortex. Annu. Rev. Neurosci. 28, 377–401. doi: 10.1146/annurev.neuro.27.070203.144216, 16022601

[ref69] Pascual-Leone A. Hamilton R. (2001). “Chapter 27 the metamodal organization of the brain,” in Progress in Brain Research, vol. 134 (Elsevier), 427–445. 11702559 10.1016/s0079-6123(01)34028-1

[ref70] Passingham R. E. Stephan K. E. Kötter R. (2002). The anatomical basis of functional localization in the cortex. Nat. Rev. Neurosci. 3, 606–616. doi: 10.1038/nrn893, 12154362

[ref71] Pelland M. Orban P. Dansereau C. Lepore F. Bellec P. Collignon O. (2017). State-dependent modulation of functional connectivity in early blind individuals. NeuroImage 147, 532–541. doi: 10.1016/j.neuroimage.2016.12.053, 28011254

[ref72] Petersen S. E. Seitzman B. A. Nelson S. M. Wig G. S. Gordon E. M. (2024). Principles of cortical areas and their implications for neuroimaging. Neuron 112, 2837–2853. doi: 10.1016/j.neuron.2024.05.008, 38834069 PMC13339775

[ref73] Petrus E. Isaiah A. Jones A. P. Li D. Wang H. Lee H.-K. . (2014). Crossmodal induction of thalamocortical potentiation leads to enhanced information processing in the auditory cortex. Neuron 81, 664–673. doi: 10.1016/j.neuron.2013.11.023, 24507197 PMC4023256

[ref74] Petrus E. Rodriguez G. Patterson R. Connor B. Kanold P. O. Lee H.-K. (2015). Vision loss shifts the balance of feedforward and intracortical circuits in opposite directions in mouse primary auditory and visual cortices. J. Neurosci. 35, 8790–8801. doi: 10.1523/JNEUROSCI.4975-14.2015, 26063913 PMC4461685

[ref75] Ptito M. Paré S. Dricot L. Cavaliere C. Tomaiuolo F. Kupers R. (2021). A quantitative analysis of the retinofugal projections in congenital and late-onset blindness. NeuroImage 32:102809. doi: 10.1016/j.nicl.2021.102809, 34509923 PMC8435915

[ref76] Ptito M. Schneider F. C. G. Paulson O. B. Kupers R. (2008). Alterations of the visual pathways in congenital blindness. Exp. Brain Res. 187, 41–49. doi: 10.1007/s00221-008-1273-4, 18224306

[ref77] Qin W. Liu Y. Jiang T. Yu C. (2013). The development of visual areas depends differently on visual experience. PLoS One 8:e53784. doi: 10.1371/journal.pone.0053784, 23308283 PMC3538632

[ref78] Raemaekers M. Schellekens W. Van Wezel R. J. A. Petridou N. Kristo G. Ramsey N. F. (2014). Patterns of resting state connectivity in human primary visual cortical areas: a 7T fMRI study. NeuroImage 84, 911–921. doi: 10.1016/j.neuroimage.2013.09.060, 24099850

[ref79] Ratan Murty N. A. Teng S. Beeler D. Mynick A. Oliva A. Kanwisher N. (2020). Visual experience is not necessary for the development of face-selectivity in the lateral fusiform gyrus. Proc. Natl. Acad. Sci. 117, 23011–23020. doi: 10.1073/pnas.2004607117, 32839334 PMC7502773

[ref80] Reich L. Szwed M. Cohen L. Amedi A. (2011). A ventral visual stream reading center independent of visual experience. Curr. Biol. 21, 363–368. doi: 10.1016/j.cub.2011.01.040, 21333539

[ref81] Reislev N. L. Dyrby T. B. Siebner H. R. Kupers R. Ptito M. (2016a). Simultaneous assessment of white matter changes in microstructure and connectedness in the blind brain. Neural Plast. 2016, 1–12. doi: 10.1155/2016/6029241, 26881120 PMC4736370

[ref82] Reislev N. H. Dyrby T. B. Siebner H. R. Lundell H. Ptito M. Kupers R. (2017). Thalamocortical connectivity and microstructural changes in congenital and late blindness. Neural Plast. 2017, 1–11. doi: 10.1155/2017/9807512, 28386486 PMC5366815

[ref83] Reislev N. L. Kupers R. Siebner H. R. Ptito M. Dyrby T. B. (2016b). Blindness alters the microstructure of the ventral but not the dorsal visual stream. Brain Struct. Funct. 221, 2891–2903. doi: 10.1007/s00429-015-1078-8, 26134685

[ref84] Ricciardi E. Vanello N. Sani L. Gentili C. Scilingo E. P. Landini L. . (2007). The effect of visual experience on the development of functional architecture in hMT+. Cereb. Cortex 17, 2933–2939. doi: 10.1093/cercor/bhm018, 17372275

[ref85] Röder B. Stock O. Bien S. Neville H. Rösler F. (2002). Speech processing activates visual cortex in congenitally blind humans. Eur. J. Neurosci. 16, 930–936. doi: 10.1046/j.1460-9568.2002.02147.x, 12372029

[ref86] Roy A. Wang S. Meschede-Krasa B. Breffle J. Van Hooser S. D. (2020). An early phase of instructive plasticity before the typical onset of sensory experience. Nat. Commun. 11:11. doi: 10.1038/s41467-019-13872-1, 31896763 PMC6940391

[ref87] Saccone E. J. Tian M. Bedny M. (2024). Developing cortex is functionally pluripotent: evidence from blindness. Dev. Cogn. Neurosci. 66:101360. doi: 10.1016/j.dcn.2024.101360, 38394708 PMC10899073

[ref88] Sen S. Khalsa N. N. Tong N. Ovadia-Caro S. Wang X. Bi Y. . (2022). The role of visual experience in individual differences of brain connectivity. J. Neurosci. 42, 5070–5084. doi: 10.1523/JNEUROSCI.1700-21.2022, 35589393 PMC9233442

[ref89] Shimony J. S. Burton H. Epstein A. A. McLaren D. G. Sun S. W. Snyder A. Z. (2006). Diffusion tensor imaging reveals white matter reorganization in early blind humans. Cereb. Cortex 16, 1653–1661. doi: 10.1093/cercor/bhj102, 16400157 PMC3789517

[ref90] Shu N. Li J. Li K. Yu C. Jiang T. (2009a). Abnormal diffusion of cerebral white matter in early blindness. Hum. Brain Mapp. 30, 220–227. doi: 10.1002/hbm.20507, 18072278 PMC6870762

[ref91] Shu N. Liu Y. Li J. Li Y. Yu C. Jiang T. (2009b). Altered anatomical network in early blindness revealed by diffusion tensor Tractography. PLoS One 4:e7228. doi: 10.1371/journal.pone.0007228, 19784379 PMC2747271

[ref92] Song S.-K. Yoshino J. Le T. Q. Lin S.-J. Sun S.-W. Cross A. H. . (2005). Demyelination increases radial diffusivity in corpus callosum of mouse brain. NeuroImage 26, 132–140. doi: 10.1016/j.neuroimage.2005.01.028, 15862213

[ref93] Stellwagen D. Shatz C. J. (2002). An instructive role for retinal waves in the development of Retinogeniculate connectivity. Neuron 33, 357–367. doi: 10.1016/S0896-6273(02)00577-9, 11832224

[ref94] Striem-Amit E. Ovadia-Caro S. Caramazza A. Margulies D. S. Villringer A. Amedi A. (2015). Functional connectivity of visual cortex in the blind follows retinotopic organization principles. Brain 138, 1679–1695. doi: 10.1093/brain/awv083, 25869851 PMC4614142

[ref95] Thiebaut De Schotten M. Forkel S. J. (2022). The emergent properties of the connected brain. Science 378, 505–510. doi: 10.1126/science.abq2591, 36378968

[ref96] Tian M. Saccone E. J. Kim J. S. Kanjlia S. Bedny M. (2023). Sensory modality and spoken language shape reading network in blind readers of braille. Cereb. Cortex 33, 2426–2440. doi: 10.1093/cercor/bhac216, 35671478 PMC10016046

[ref97] Tian M. Xiao X. Hu H. Cusack R. Bedny M. (2026). Visual experience shapes functional connectivity between occipital and non-visual networks. eLife 13:RP93067. doi: 10.7554/eLife.93067, 41848400 PMC12999175

[ref98] van den Hurk J. Van Baelen M. Op de Beeck H. P. (2017). Development of visual category selectivity in ventral visual cortex does not require visual experience. Proc. Natl. Acad. Sci. 114, E4501–E4510. doi: 10.1073/pnas.1612862114, 28507127 PMC5465914

[ref99] Wandell B. A. Dumoulin S. O. Brewer A. A. (2007). Visual field maps in human cortex. Neuron 56, 366–383. doi: 10.1016/j.neuron.2007.10.012, 17964252

[ref100] Wang X. He C. Peelen M. V. Zhong S. Gong G. Caramazza A. . (2017). Domain selectivity in the parahippocampal gyrus is predicted by the same structural connectivity patterns in blind and sighted individuals. J. Neurosci. 37, 4705–4716. doi: 10.1523/JNEUROSCI.3622-16.2017, 28381591 PMC6596493

[ref101] Wang X. Peelen M. V. Han Z. Caramazza A. Bi Y. (2016). The role of vision in the neural representation of unique entities. Neuropsychologia 87, 144–156. doi: 10.1016/j.neuropsychologia.2016.05.007, 27174518

[ref102] Wang X. Peelen M. V. Han Z. He C. Caramazza A. Bi Y. (2015). How visual is the visual cortex? Comparing connectional and functional fingerprints between congenitally blind and sighted individuals. J. Neurosci. 35, 12545–12559. doi: 10.1523/JNEUROSCI.3914-14.2015, 26354920 PMC6605405

[ref103] Wang D. Qin W. Liu Y. Zhang Y. Jiang T. Yu C. (2013). Altered white matter integrity in the congenital and late blind people. Neural Plast. 2013, 1–8. doi: 10.1155/2013/128236, 23710371 PMC3654351

[ref104] Watkins K. E. Cowey A. Alexander I. Filippini N. Kennedy J. M. Smith S. M. . (2012). Language networks in anophthalmia: maintained hierarchy of processing in “visual” cortex. Brain 135, 1566–1577. doi: 10.1093/brain/aws067, 22427328

[ref105] Wen Z. Kang Y. Zhang Y. Yang H. Xie B. (2022). Alteration of degree centrality in adolescents with early blindness. Front. Hum. Neurosci. 16:935642. doi: 10.3389/fnhum.2022.935642, 35832871 PMC9271564

[ref106] Wolbers T. Klatzky R. L. Loomis J. M. Wutte M. G. Giudice N. A. (2011a). Modality-independent coding of spatial layout in the human brain. Curr. Biol. 21, 984–989. doi: 10.1016/j.cub.2011.04.038, 21620708 PMC3119034

[ref107] Wolbers T. Zahorik P. Giudice N. A. (2011b). Decoding the direction of auditory motion in blind humans. NeuroImage 56, 681–687. doi: 10.1016/j.neuroimage.2010.04.266, 20451630

[ref108] Xu Y. Vignali L. Sigismondi F. Crepaldi D. Bottini R. Collignon O. (2023). Similar object shape representation encoded in the inferolateral occipitotemporal cortex of sighted and early blind people. PLoS Biol. 21:e3001930. doi: 10.1371/journal.pbio.3001930, 37490508 PMC10368275

[ref109] Yu C. Liu Y. Li J. Zhou Y. Wang K. Tian L. . (2008). Altered functional connectivity of primary visual cortex in early blindness. Hum. Brain Mapp. 29, 533–543. doi: 10.1002/hbm.20420, 17525980 PMC6871175

[ref110] Yu C. Shu N. Li J. Qin W. Jiang T. Li K. (2007). Plasticity of the corticospinal tract in early blindness revealed by quantitative analysis of fractional anisotropy based on diffusion tensor tractography. NeuroImage 36, 411–417. doi: 10.1016/j.neuroimage.2007.03.003, 17442594

[ref111] Zhang F. Daducci A. He Y. Schiavi S. Seguin C. Smith R. E. . (2022). Quantitative mapping of the brain’s structural connectivity using diffusion MRI tractography: a review. NeuroImage 249:118870. doi: 10.1016/j.neuroimage.2021.118870, 34979249 PMC9257891

[ref112] Zhou Z. Qian L. Xu J. Lu Y. Hou F. Zhou J. . (2022). Topologic reorganization of white matter connectivity networks in early-blind adolescents. Neural Plast. 2022, 1–11. doi: 10.1155/2022/8034757, 35529452 PMC9072039

